# From Personal Care to Coastal Concerns: Investigating Polyethylene Glycol Impact on Mussel’s Antioxidant, Physiological, and Cellular Responses

**DOI:** 10.3390/antiox13060734

**Published:** 2024-06-17

**Authors:** Cristiana Roberta Multisanti, Giorgia Zicarelli, Alessia Caferro, Mariacristina Filice, Caterina Faggio, Irene Vazzana, Jana Blahova, Pavla Lakdawala, Maria Carmela Cerra, Sandra Imbrogno, Federica Impellitteri

**Affiliations:** 1 Department of Veterinary Sciences, University of Messina, 98168 Messina, Italy; cristiana.multisanti@studenti.unime.it (C.R.M.); federica.impellitteri@studenti.unime.it (F.I.); 2 Department of Chemical, Biological, Pharmaceutical and Environmental Sciences, University of Messina, 98166 Messina, Italy; giorgia.zicarelli@studenti.unime.it (G.Z.); caterina.faggio@unime.it (C.F.); 3 Department of Biology, Ecology and Earth Science, University of Calabria, 87036 Rende, Italy; alessia.caferro@unical.it (A.C.); maria_carmela.cerra@unical.it (M.C.C.); 4 Department of Ecosustainable Marine Biotechnology, Stazione Zoologica Anton Dohrn, 80122 Naples, Italy; 5 Zooprophylactic Institute of Sicily, Via Gino Marinuzzi, 90129 Palermo, Italy; irene.vazzana@izssicilia.it; 6 Department of Animal Protection and Welfare & Veterinary Public Health, Faculty of Veterinary Hygiene and Ecology, University of Veterinary Sciences Brno, 612 42 Brno, Czech Republic; blahovaj@vfu.cz (J.B.); lakdawalap@vfu.cz (P.L.)

**Keywords:** mussels, gills, digestive gland, aquatic pollution, personal care products

## Abstract

Pharmaceutical and personal care products (PPCPs) containing persistent and potentially hazardous substances have garnered attention for their ubiquitous presence in natural environments. This study investigated the impact of polyethylene glycol (PEG), a common PPCP component, on *Mytilus galloprovincialis*. Mussels were subjected to two PEG concentrations (E1: 0.1 mg/L and E2: 10 mg/L) over 14 days. Oxidative stress markers in both gills and digestive glands were evaluated; cytotoxicity assays were performed on haemolymph and digestive gland cells. Additionally, cell volume regulation (RVD assay) was investigated to assess physiological PEG-induced alterations. In the gills, PEG reduced superoxide dismutase (SOD) activity and increased lipid peroxidation (LPO) at E1. In the digestive gland, only LPO was influenced, while SOD activity and oxidatively modified proteins (OMPs) were unaltered. A significant decrease in cell viability was observed, particularly at E2. Additionally, the RVD assay revealed disruptions in the cells subjected to E2. These findings underscore the effects of PEG exposure on *M. galloprovincialis*. They are open to further investigations to clarify the environmental implications of PPCPs and the possibility of exploring safer alternatives.

## 1. Introduction

Emerging contaminants (ECs) are gaining particular attention due to their increasing prevalence in natural ecosystems, driving the interest in understanding their contribution to environmental pollution [1,2,3,4]. Especially in today’s post-pandemic period, the extent of COVID-19, the strategies employed to manage the disease, and the treatments administered to patients have led to significant changes in the production and use of individual protective equipment, pharmaceuticals, personal care/hygiene products, and disinfectants, with consequent impact on their release and distribution in the environment [5,6,7]. Being pseudo-persistent pollutants, pharmaceutical and personal care products (PPCPs) are hardly or even not removed by wastewater treatment systems [8,9]. Particularly, personal care products (PCPs) encompass a wide range of items intended for personal hygiene, grooming, and cosmetic purposes. PPCP components can vary depending on their use and formulation. Common ingredients found in PPCPs include surfactants, emollients, preservatives, fragrances, UV filters, etc. [10,11,12]. Some of these components exhibit characteristics such as persistence and bioaccumulation, posing potential health risks to the environment and living organisms [8,13].

Polyethylene glycols (PEGs) are polymers increasingly incorporated into a wide variety of PPCPs. Additionally, PEG serves as a stabiliser for lipid nanoparticles in mRNA vaccines, such as the Pfizer/BioNTech. In this context, Sellaturay et al. [14] demonstrated that a PEG allergy can lead to anaphylaxis following vaccination. Although PEG is included as an excipient in many drug formulations to improve their pharmacokinetic properties [15], the likelihood of hypersensitive reactions may vary according to its molecular weight, and, although rare, allergic responses to PEGs can be severe.

The global production of PEG reaches millions of tons annually [16]. Although precise data on the presence of PEG and its derivatives in surface water are lacking, Traverso-Soto et al. [17] stated that concentrations in wastewater may exceed 1 mg/L. Consequently, a notable quantity of PEG in surface waters has been observed to impact the bioavailability and toxicity of other environmental pollutants. Studies generally indicate a low toxicity of PEG in organisms [18,19,20]. Nevertheless, instances of nephrotoxicity [15] and reports of adverse effects, such as damage to the central nervous system, heart, and lungs, as well as kidney failure, have been documented in ethylene glycol-treated subjects [21]. In addition, in common carp (*Cyprinus carpio*), Hatami et al. [22] observed disruptions in cell membranes after PEG exposure. 

For the reasons mentioned above, the main purpose of the present study was to further investigate PEG-induced toxicity in marine non-target organisms, since aquatic ecosystems represent one of the major sinks of ECs. The Mediterranean mussel *(Mytilus galloprovincialis*) has been chosen as a model due to its biological features, such as widespread distribution, bioindicator capabilities, filter-feeding mechanism, resilience, etc., as well as for its suitable response to anthropogenic pressures [23,24,25]. The effects of PEG exposure were assessed by examining the potential physiological, antioxidant, and cellular responses in the mussels’ haemolymph, gills, and digestive gland (DG), as they represent the first line of defence (haemocytes and gills) and the primary detoxifying organ (DG). Potential alterations were analysed by investigating the viability of the haemolymph and DG cells, as well as the ability of hepatocytes to regulate their cellular volume. In aquatic organisms, the response of the antioxidant system is crucial for evaluating the effects of xenobiotics [26]. In this context, biochemical enzymatic and non-enzymatic parameters are widely used as suitable endpoints of toxicity [27]. Consequently, in both gills and DGs, superoxide dismutase (SOD) activity, lipid peroxidation, and carbonylated proteins content have been evaluated. The results of the present study may provide comprehensive knowledge of the adverse effects of PEGs on marine bivalve molluscs, as well as useful indications to further analyse a wider range of damage caused by the persistence of PEGs to aquatic communities, finally providing useful information on the correct use of PEG-based compounds.

## 2. Materials and Methods

### 2.1. Experimental Design 

The toxicity test was performed on one hundred fifty specimens of *M. galloprovincialis,* purchased from a mussel commercial farm, FARAU S.r.l., Frutti di Mare, located in the “Lago Faro” in the reserve of “Capo Peloro Lagoon” (Messina, Italy). *M. galloprovincialis* samples, with a mean length of 5.5 ± 0.2 cm (expressed as the mean length ± S.E.), were rapidly and promptly transported to the Laboratory of Animals Ecophysiology at the University of Messina. The specimens were randomly divided into six aquariums containing 20 L of brackish, filtered lake water provided by the same mussel farm. Before the start of the experiment, the samples were acclimated for a week with continuous oxygen aeration and a water change every two days. The concentration of PEG was measured before and after every change of the water. 

The animals were then divided into three groups (ctrl 0 mg/L, PEG1 0.1 mg/L, and PEG2 10 mg/L) in duplicate. The experimental design is illustrated in Table 1. Throughout the 14-day experimental time, the animals were kept under the standard conditions of salinity (3.4 ± 0.2%), pH (7.6 ± 0.01), and temperature (18.0 ± 0.2 °C). The daily photoperiod was maintained at 12 h of light and 12 h of darkness. The water was changed three times per week with brackish water enriched with nutrients and continuous aeration was kept. 

PEG (CAS number 25322-68-3) was purchased from Sigma-Aldrich (Prague, Czech Republic). The chemical is presented in powder form (99% purity) with a size of 8000 and is dissolved in brackish lake water without using any solvent. The size was selected following the results obtained by Zicarelli et al. [28]. Due to a lack of information about the exact concentration in the environment, the concentrations used in the experiments were chosen based on previous studies on PEG [17,22,28]. 

### 2.2. Collection of Samples and Cell Viability Assay

The haemolymph samples were collected from the adductor muscle of four randomly selected animals by using a syringe with a 5 cm needle. DG cells were isolated using the method described in detail by Impellitteri et al. [23]. The haemolymph and DG cells were used to evaluate cell viability. 

Two different colorimetric tests were used to assess cell viability: the Neutral Red retention assay (NR) and the Trypan Blue exclusion method (TB). In the NR assay, the haemolymph and DG cells were treated with a 0.8% NR solution diluted 1:1000 and kept incubated with the samples for 10 min following the protocol described by Moore et al. [29]. The cells were considered viable if their lysosomal membranes, observed under a light microscope at 40× magnification, remained intact and red-coloured. In the TB assay, the cells were incubated in a 1:1 solution of TB at 4% for 5 min, and viability was calculated using the formula described by Tresnakova et al. [30]. Since TB is unable to penetrate the membranes of living cells, all blue-stained cells were considered non-viable. The tests were conducted using Bürker and Neubauer chambers purchased from Fisher Scientific (Hempton, NH, USA). 

### 2.3. Regulation Volume Decrease (RVD) 

The RVD test was performed by placing a drop of DG cell samples on a slide treated with polylysine to facilitate cell adhesion. The samples were then observed using a Carl Zeiss Axioskop 20 (Wetzlar, Germany) light microscope at 100× magnification connected to a Canon 550D digital camera. For the RVD analyses, the samples were washed with an isotonic solution (1100 mOsm) containing NaCl 550 mM, KCl 12.5 mM, MgSO_4_ 8 mM, CaCl_2_ 4 mM, glucose 10 mM, MgCl_2_ 40 mM, and HEPES 20 mM. Three photos were taken in succession; then, the slide was washed with a hypotonic solution (800 mOsm) containing NaCl 350 mM, KCl 12.5 mM, MgSO_4_ 8 mM, CaCl_2_ 4 mM, glucose 10 mM, MgCl_2_ 40 mM, and HEPES 20 mM. Seventeen photos were taken in total: ten photos were taken over ten minutes (one per minute from IPO1 to IPO10), and four photos were taken over the following twenty minutes (one photo every five minutes from IPO11 to IPO14). Fifteen cells were selected from each experimental group, and the cell area was calculated using the ImageJ software Version 1.54i (NIH, Bethesda, MD, USA) for comparison between the cell area of the control group and the exposed animals.

### 2.4. Biochemical Parameters 

The electrolytes present in the haemolymph and the aquarium water were evaluated for each experimental group using the multi-parametric analyser KONELAB 60 THERMO (Milan, Italy). The percentages of Na^+^, K^+^, Cl^−^, P, Mg^2+^, and Ca^2+^ were measured. Additionally, haemolymph lactate dehydrogenase (LDH) was used as a marker of cell damage.

### 2.5. Oxidative Markers

The evaluation of oxidative stress biomarkers was performed as described by Filice et al. [31]. The gills and digestive glands (N = 6 for each condition) were homogenised in cold 100 mM Tris/HCl buffer (pH 7.2) (Sigma Aldrich, Milan, Italy) containing a mixture of protease inhibitors. An aliquot of the homogenate was used to determine lipid peroxidation; the remaining part was centrifuged at 5000× *g* for 5 min at 4 °C and the supernatant was used to analyse both protein oxidation and superoxide dismutase (SOD) activity. Protein concentration in the supernatant was determined according to the Bradford method by using a commercial kit (Bio-Rad Laboratories, Hercules, CA, USA) and bovine serum albumin (BSA) as a standard.

#### 2.5.1. Lipid Peroxidation

Lipid peroxidation (LPO) was assessed by measuring the concentration of 2-thiobarbituric acid-reacting substances (TBARS) according to Tkachenko and Grudniewska [32]. A reaction mixture containing the sample homogenate (0.2 mL, 10% *w*/*v*) in Tris/HCl buffer (100 mM, pH 7.2), 2-thiobarbituric acid (TBA; 0.8%, 0.2 mL), and trichloroacetic acid (TCA; 20%, 0.2 mL) was kept in a water bath at 100 °C for 10 min and then centrifuged at 7000 rpm for 10 min. The supernatant was assessed at 540 nm to determine malondialdehyde (MDA) content, the major lipid oxidation product. The TBARS values were expressed as MDA concentration (nM) per gram of tissue (MDA extinction coefficient: 156,000 M^−1^ cm^−1^). All reagents were from Sigma Aldrich (Milan, Italy).

#### 2.5.2. Protein Oxidation

The oxidatively modified protein (OMP) levels were evaluated by measuring carbonyl group content performed through the traditional 2,4-dinitrophenylhydrazine (DNPH) method described by Levine et al. [33]. Aliquots of the supernatant were incubated at room temperature for 1 h with 10 mM DNPH (Sigma Aldrich, Milan, Italy) in 2 M HCl and then precipitated with 2 volumes of TCA. The solution was centrifuged for 20 min at 7000 rpm; the pellet was washed thrice with ethanol–ethyl acetate (1:1; *v/v*) to remove DNPH excess and then dissolved in 6 M guanidine in 2 N HCl. The concentration of carbonyl groups was measured spectrophotometrically at 370 nm (aldehydic derivatives) and at 430 (ketonic derivatives) using the extinction coefficient of 22,000 M^−1^ cm^−1^. The results were expressed as nmol per mg protein.

#### 2.5.3. SOD Activity

The SOD activity was determined by the pyrogallol method of Marklund and Marklund [34], modified by Filice et al. [35]. In brief, the inhibitory effect of SOD on the auto-oxidation of pyrogallol at pH 8.20 was assayed spectrophotometrically at 420 nm and 25 °C. The reaction was run in 50 mM Tris-HCl, 1 mM EDTA, and 0.2 mM pyrogallol (Thermo Fisher Scientific Inc., Milan, Italy) and monitored every 30 s for 5 min. One unit of SOD activity was defined as the amount of the enzyme that inhibits 50% of pyrogallol auto-oxidation. The results were expressed in U/mg protein.

### 2.6. Data Analyses 

After checking the normality and homogeneity of the data using the Kolmogorov–Smirnov and Levene tests, the analysis of variance (one-way ANOVA), followed by the Tuckey post hoc tests for comparison, was applied to analyse the results. The statistical analyses were performed using the statistical software GraphPad Prism, Version 8.2.1. (GraphPad Software Ltd., La Jolla, CA, USA). Significant results were considered with a *p*-value < 0.05. The results are presented as mean ± standard error (S.E.).

## 3. Results

### 3.1. Cell Viability 

Table 2 shows the haemocyte viability. The TB exclusion test revealed significant differences in the cells from the PEG2-treated animals (94.3%) compared to the control (99%, *p* < 0.05). However, no significant differences were found between the control and treated animals when the NR retention test was used. 

TB staining showed that DG cell viability was significantly reduced in the PEG2-treated animals (93.3%) compared to the control (98.7%, *p* < 0.01). When tested with the NR assay, the DG cells exposed to PEG1 (97.3%) showed a significant reduction in viability compared to the control (99.4%, *p* < 0.05). The results are summarised in Table 3.

### 3.2. Regulation Volume Decrease 

**Figure 1:**
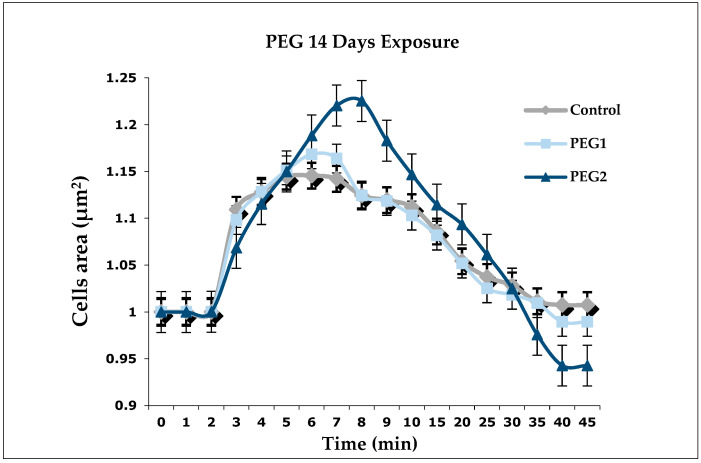
Regulation of volume decrease (RVD) in the digestive gland cells of *M. galloprovincialis* (n = 6) exposed to two different concentrations of PEG for 14 days. Rhombuses (◆) represent control (0 mg/L), square (■) represents PEG1 (0.1 mg/L), and triangles (▴) represent PEG2 (10 mg/L). The values are the mean ± S.E. of the selected cells (n = 15). The analyses were made using a one-way ANOVA test; no significant differences were highlighted.

Figure 1 shows the results of the 14-days exposure on the DG cells. No significant differences were observed in PEG1 and PEG2 compared to the control. In the control groups, the cells reached the maximum swelling in IPO4 and showed the ability to restore their initial volume. However, the cells exposed to PEG1 reached the peak of swelling around IPO5 and the cells exposed to PEG2 reached their maximum swell around IPO6. Nevertheless, the cells belonging to the PEG2 group showed the inability to return to their original volume. Although the cells of PEG1 and PEG2 took longer to reach their peak compared to the control, the cells exposed to PEG1 increased their volume by 2.3% more than the control cells, and those exposed to PEG2 increased their volume by approximately 8% more than the control cells.

### 3.3. Biochemical Parameters 

The biochemical parameters of the haemolymph did not show any significant differences between the control and experimental groups. However, as shown in Table 4, the values of Na^+^, K^+^, Cl^−^, and Ca^2+^ increased in the experimental groups. 

The amount of electrolytes dissolved into the water did not show significant differences among the groups. In particular, no significant changes in the values of Na^+^, K^+^, Cl^−^, Mg^2+^, and Ca^2+^ were observed in the water of both the experimental groups. The results are summarised in Table 5.

### 3.4. Oxidative Status Evaluation

The activity of the SOD enzyme and the levels of LPO and OMP, as indexes of the oxidative status, were measured in the gills and DG of *M. galloprovincialis* exposed to PEG. 

**Figure 2:**
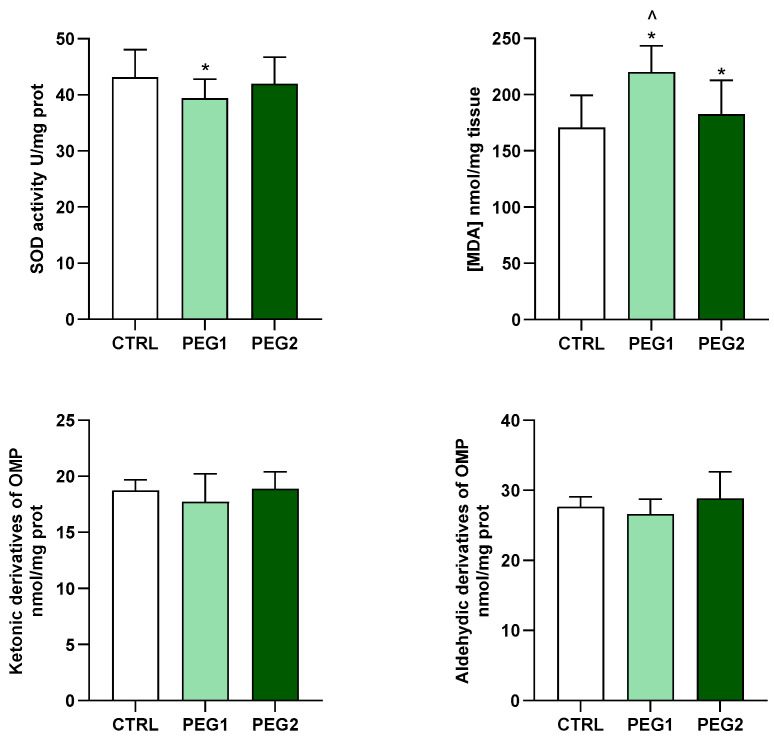
SOD activity, TBARS, and OMP in the gills of *M. galloprovincialis* exposed to PEG. The data are expressed as mean ± S.E. of absolute values (n = 6) of the individual experiments performed in duplicate. Statistics were assessed by one-way ANOVA followed by Tukey’s multiple comparison test (*p* < 0.05 * CTRL vs. PEG1 or PEG2; ^ PEG1 vs. PEG2).

In the gills, the activity of SOD was significantly reduced in the mussels exposed to the low concentration of PEG, while it showed values similar to the control group in the animals exposed to the highest concentrations tested. Compared to the control group, LPO, measured in terms of TBARS levels, increased in response to PEG exposure at both the concentrations tested. However, at the E2 concentration, the TBARS levels were lower than those in E1. No significant differences in both aldehydic and ketonic derivatives (OMP) were observed in the three experimental conditions (Figure 2).

**Figure 3:**
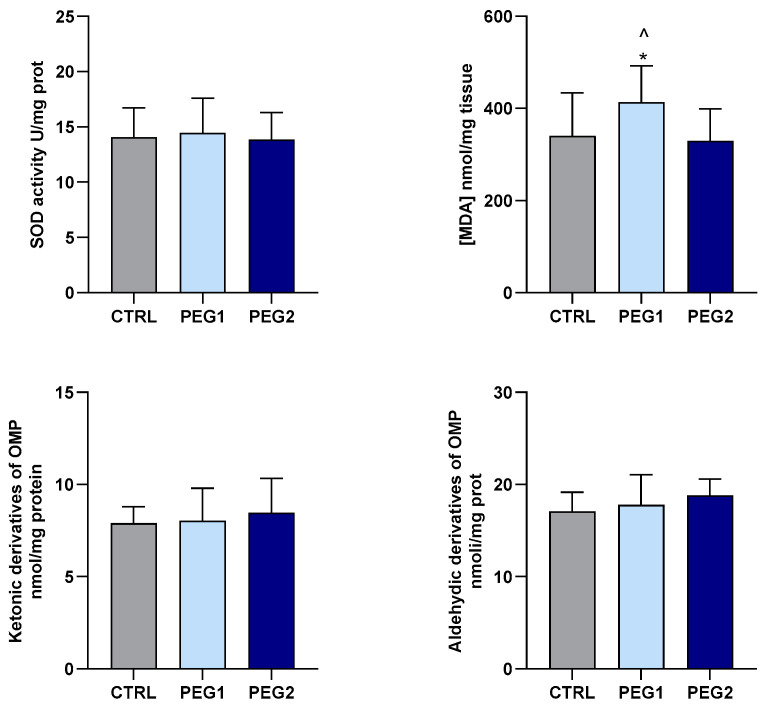
SOD activity, TBARS, and OMP in the DG of *M. galloprovincialis* exposed to PEG. The data are expressed as mean ± S.E. of absolute values (n = 6) of the individual experiments performed in duplicate. Statistics were assessed by one-way ANOVA followed by Tukey’s multiple comparison test (*p* < 0.05 * CTRL vs. PEG1; ^ PEG1 vs. PEG2).

In the DG, exposure to PEG only affected LPO. Indeed, a significant increase in TBARS levels was observed in the mussels exposed to the lowest concentration tested. No differences in SOD activity, nor in OMP levels, were observed among the groups (Figure 3).

## 4. Discussion

To investigate the toxicity of potentially toxic compounds contained in PPCP formulations, such as PEG, in aquatic ecosystems, *M. galloprovincialis* has proven to be a suitable sentinel organism [24,36,37] and the use of haemolymph, DG cells, biochemical parameters, and oxidative stress resulting in being suitable endpoints for toxicological investigations [38,39]. Our investigation showed a significant interaction between PEG and *M. galloprovincialis*, culminating in a notable decrease in the vitality of both haemocytes and hepatocytes. These cell populations play key roles in mussel’s physiological processes, including immune response, metabolism, and detoxification [40,41]. The observed decline in cell viability is evident in the NR and TB assays. The results related to the NR retention assay suggest the impairment of lysosomal membranes. Lysosomes, integral to the organism’s defence mechanisms, are crucial for maintaining cellular homeostasis and responding to external stressors [30,38]. Indeed, mussels exhibit remarkable resilience in coping with harsh and contaminated environments, partly owing to their ability to regulate lysosomal activity. Lysosomes, through their autophagic function, serve as the organism’s initial line of defence, enabling the degradation and recycling of cellular components to mitigate the impact of xenobiotics and pathogens [30,38]. Therefore, any deviation from the normal regulatory mechanisms of lysosomal activity serves as a sensitive indicator of cytotoxicity, reflecting the organism’s compromised ability to maintain cellular homeostasis [2,8]. In addition, under physiological conditions, healthy cell membranes do not allow TB to enter the cell. In contrast, in the present study, the significant results of TB exclusion in the cell membrane of both the haemocytes and DG cells suggest the likelihood of the impairment of cell membrane integrity as a consequence of exposure to the highest concentration of PEG (10 mg/L). Interestingly, our findings resonate with those reported by Hatami et al. [22], who observed similar disruptions in cell membranes in common carp (*Cyprinus carpio*) exposed to PEG at a concentration of 10 mg/L.

In the present study, the *M. galloprovincialis* specimens were found to be affected by exposure to different concentrations of PEG. These findings highlighted the negative interaction between PEG and DG cells since this product impairs cell ability to regulate the volume. The ability of DG cells to regulate their volume when subjected to osmotic changes in physiological conditions has been widely investigated [42,43]. In this context, the RVD analysis highlights how this mechanism could be altered by long-term exposure to xenobiotics. Our findings are in line with previous studies conducted on mussels exposed to chemicals involved in PPCP production [23,30,38]. The loss of the ability to regulate cell volume is a sign of alteration in the cytoskeleton, damage to the protein channels that alters the physiological function of cells, and cell membranes [44,45]. 

In the present study, electrolyte evaluation showed that the ion content of the haemolymph was slightly affected by PEG, although the changes were not statistically significant. Further analyses, carried out at different and longer exposure times, are needed to better clarify this aspect. Our data are of relevance in relation to mussels’ osmoregulatory capabilities that allow them to regulate internal osmolarity in relation to the surrounding aquatic environment [23]. In general, shifts in electrolyte compositions serve as indicators of mussel’s health status. In fact, haemolymph contains essential constituents such as Cl^−^, Na^+^, K^+^, Ca^2+^, Mg^2+^, etc., which play roles in diverse physiological functions, including metabolism, enzymatic activities, shell development, osmoregulation, and the maintenance of the organism’s internal balance [30].

In aquatic species, exposure to pollutants results in an alteration of oxidative homeostasis and a consequent increase in the production of reactive oxygen species (ROS). ROS can easily interact with macromolecules (i.e., DNA, protein, and lipid) leading to structural and functional changes that can be detrimental to animal fitness [46,47]. In this context, the analysis of well-established biological markers represents a valuable tool for assessing the severity of these events. Among others, the activity of antioxidant enzymes and the levels of oxidation products (TBARS and OMP) are typically used to detect changes in the oxidative status of the whole organism and of specific target tissues [31,45,48,49]. A PPCP-dependent modulation of oxidative biomarkers has been reported in several aquatic species, including *Carassius auratus* [50] and *Oreochromis niloticus* [51]. Data about the influence of PEG on the oxidative status of aquatic animals are scarce. The few available data mainly refer to *C. carpio*, in which 21 days of exposure to 5 and 10 mg/L of PEG did not affect acetylcholinesterase (AChE) activity in plasma, and neither CAT activity nor lipid peroxidation in the liver [22]. In our study, we observed that 14 days of exposure to 10 mg/L of PEG did not affect the oxidative status of *M. galloprovincialis* in the DG and gill tissues, with the exception of a slight significative increase in lipid peroxidation in the gills. On the contrary, exposure to the lowest PEG concentration (0.1 mg/L) negatively affected the activity of the SOD enzyme in the gills, while increasing lipid peroxidation in both tissues. These data are preliminary and require that other enzymes involved in the antioxidant response are investigated to fully describe mussel’s oxidative status in the presence of PEG. However, the information obtained on SOD (in the gills) and on the levels of oxidation products (in both gills and DG) suggest that in *M. galloprovincialis*, a low concentration of PEG may inhibit the antioxidant defence system. This, by limiting the capacity to eliminate ROS, may result in lipid oxidative damage. The tissue-specific response that we observed is not surprising, since in a mussel the gills represent the first tissue to be exposed to water contaminants. We cannot provide a conclusive explanation concerning the absence of effects observed on SOD activity at the highest PEG concentration, particularly in DG. It is possible that the activation of other mechanisms of protection against ROS contributes to preserving the redox balance. Although caution is needed when comparing the effects of different chemicals, it is intriguing that a similar concentration-dependent behaviour has been observed in *M. galloprovincialis* following exposure to other PCP components. This is the case of Sodium Lauryl Sulphate which was found to affect the activity of antioxidant enzymes only at the lowest concentrations tested [52].

## 5. Conclusions

In light of the increasing prevalence of emerging contaminants like PEG in aquatic environments, understanding their toxicological effects on marine organisms is imperative. This study aimed to investigate the impact of PEG exposure on *M. galloprovincialis*, serving as a crucial sentinel organism for assessing ecological health. Through comprehensive analyses encompassing cell viability, the regulation of volume decrease (RVD), biochemical parameters, and oxidative stress, this study sheds light on the adverse effects of PEG on cellular physiology and homeostasis in *M. galloprovincialis.* This contributes to advancing our understanding of the ecological impact of emerging contaminants and informs strategies for sustainable environmental management. Further investigations are warranted to delve deeper into the complex interactions between PEG and marine organisms, paving the way for informed conservation efforts and ecosystem protection.

## Figures and Tables

**Table 1:** Experimental design.

Experimental Groups	Number of Specimens per Tank	Replicates	Total of Specimens per Group
Control (ctrl)	25	2	50
PEG1 (0.1 mg/L of PEG)	25	2	50
PEG2 (10 mg/L of PEG)	25	2	50

**Table 2:** Viability of the haemolymph cells of *M. galloprovincialis* (n = 6) exposed for 14 days to PEG. The tests conducted were the TB exclusion method and the NR retention assay.

Haemocytes Viability			
	Ctrl	PEG1	PEG2
Assays			
TB	99.0 ± 0.1	97.2 ± 1.0	94.3 ± 1.0 **
NR	99.9 ± 0.6	97.6 ± 1.1	97.4 ± 1.0

The results are expressed as the percentage of the mean ± S.E. from 10 slides. Significant differences compared to the control are indicated by ** *p* < 0.01. The analyses were performed using a one-way ANOVA test.

**Table 3:** Viability of the DG cells of *M. galloprovincialis* (n = 6) exposed for 14 days to PEG. The tests conducted were the TB exclusion method and the NR retention assay.

DG Cells Viability			
	Ctrl	PEG1	PEG2
Assays			
TB	98.7 ± 0.2	96.9 ± 1.0	93.3 ± 1.1 **
NR	99.4 ± 0.3	97.3 ± 0.5 *	97.9 ± 0.7

The results are expressed as the percentage of the mean ± S.E. from 10 slides. Significant differences compared to the control are indicated by * *p* < 0.05 and ** *p* < 0.01. The analyses were performed using a one-way ANOVA test.

**Table 4:** Biochemical characteristics of haemolymph of *M. galloprovincialis* (n = 6) exposed to two different concentrations of PEG (0.1 mg/L and 10 mg/L) for 14 days.

Haemolymph Biochemical Parameters			
	Ctrl	PEG1	PEG2
**Na^+^**	476 ± 22 mmol/L	503 ± 10 mmol/L	524 ± 7.0 mmol/L
**K^+^**	12 ± 0.7 mmol/L	12 ± 0.5 mmol/L	12 ± 0.2 mmol/L
**Cl^−^**	516 ± 21 mmol/L	543 ± 19 mmol/L	563 ± 0.5 mmol/L
**P**	0.8 ± 0.1 mg/dL	1.7 ± 0.1 mg/dL	2 ± 0.2 mg/dL
**Mg^2+^**	116 ± 3.0 mg/dL	114 ± 3.0 mg/dL	117 ± 0.4 mg/dL
**Ca^2+^**	45 ± 4.0 mg/dL	47 ± 0.6 mg/dL	50 ± 0.1 mg/dL
**LDH**	2.0 ± 0.0 U/L	2.0 ± 0.0 U/L	1.5 ± 0.5 U/L

The results are expressed as mean ± S.E. The analyses were performed using a one-way ANOVA test. No statistical differences have been observed.

**Table 5:** Biochemical parameters of the water from both the control and PEG1 and PEG2 experimental tanks.

Water Biochemical Parameters			
	Ctrl	PEG1	PEG2
**Na^+^**	477 ± 65 mmol/L	513 ± 18 mmol/L	530 ± 26 mmol/L
**K^+^**	10 ± 1.3 mmol/L	10 ± 0.3 mmol/L	11 ± 5.4 mmol/L
**Cl^−^**	510 ± 70 mmol/L	560 ± 18 mmol/L	567 ± 34 mmol/L
**P**	5 ± 1.9 mg/dL	7 ± 4.1 mg/dL	1.5 ± 1.2 mg/dL
**Mg^2+^**	110 ± 9.0 mg/dL	124 ± 3.0 mg/dL	122 ± 31 mg/dL
**Ca^2+^**	41 ± 8.6 mg/dL	47 ± 0.7 mg/dL	50 ± 2.9 mg/dL

The results are expressed as mean ± S.E. The analyses were performed using a one-way ANOVA test.

## Data Availability

Data are contained within the article.
